# Computer-aided drug design for virtual-screening and active-predicting of main protease (M^pro^) inhibitors against SARS-CoV-2

**DOI:** 10.3389/fphar.2023.1288363

**Published:** 2023-11-07

**Authors:** Renhui Dai, Hongwei Gao, Ruiling Su

**Affiliations:** School of Life Science, Ludong University, Yantai, Shandong, China

**Keywords:** SARS-CoV-2, M pro inhibitors, virtual screening, molecular docking, molecular dynamic simulation

## Abstract

**Introduction:** SARS-CoV-2 is a novel coronavirus with highly contagious and has posed a significant threat to global public health. The main protease (M^pro^) is a promising target for antiviral drugs against SARS-CoV-2.

**Methods:** In this study, we have used pharmacophore-based drug design technology to identify potential compounds from drug databases as M^pro^ inhibitors.

**Results:** The procedure involves pharmacophore modeling, validation, and pharmacophore-based virtual screening, which identifies 257 compounds with promising inhibitory activity.

**Discussion:** Molecular docking and non-bonding interactions between the targeted protein M^pro^ and compounds showed that ENA482732 was the best compound. These results provided a theoretical foundation for future studies of M^pro^ inhibitors against SARS-CoV-2.

## 1 Introduction

Severe Acute Respiratory Syndrome coronavirus 2 (SARS-CoV-2) is a novel coronavirus that is highly contagious and poses a significant threat to global public health (Chan et al., 2020; Scheen et al., 2020). In December 2019, this disease was initially started in the local seafood market in Wuhan, China, and then spread across the globe rapidly in a catastrophic effect. The disease is characterized by severe respiratory disorders having flu-like symptoms, such as sore throat, fever, dry cough, shortness of breath, and severe pneumonia (CSGotICoTo, 2020; Dinda et al., 2022). Existing data show that, in addition to the respiratory system, SARS-CoV-2 can also cause disease symptoms in the cardiovascular system, nervous system, gastrointestinal tract, and even eyes, and in critical cases, that lead to several organ failures and ultimately death (Hassan et al., 2021). The possible main route of transmission is thought to be close contact and respiratory droplets secreted by the patient during coughing, sneezing, breathing and even normal speech and studies have shown that the virus may also be transmitted via the fecal-oral route (Falahi and Kenarkoohi, 2020; Iwamoto et al., 2022; Targoński et al., 2022).

The COVID-19 pandemic is almost over. However, effective treatments and drugs for the disease have yet to emerge. Although many countries have successfully developed new coronavirus vaccines, the number of new infections worldwide steadily increases daily (Duan et al., 2022; Espeseth et al., 2022). At present, active prevention and isolation works are the most basic ways and means for people to deal with the epidemic. However, with the continuous development of the epidemic, new pathogenic characteristics have emerged one after another, and most patients have no apparent symptoms at the initial stage of infection or are even asymptomatic, which makes it more difficult to control the large-scale outbreak of the epidemic by isolating patients (van der Toorn et al., 2021). This situation has intensified the urgency of effective drug research and development, and finding effective drugs is one of the main focus points in the current pharmaceutical research and development field.

**FIGURE 1:**
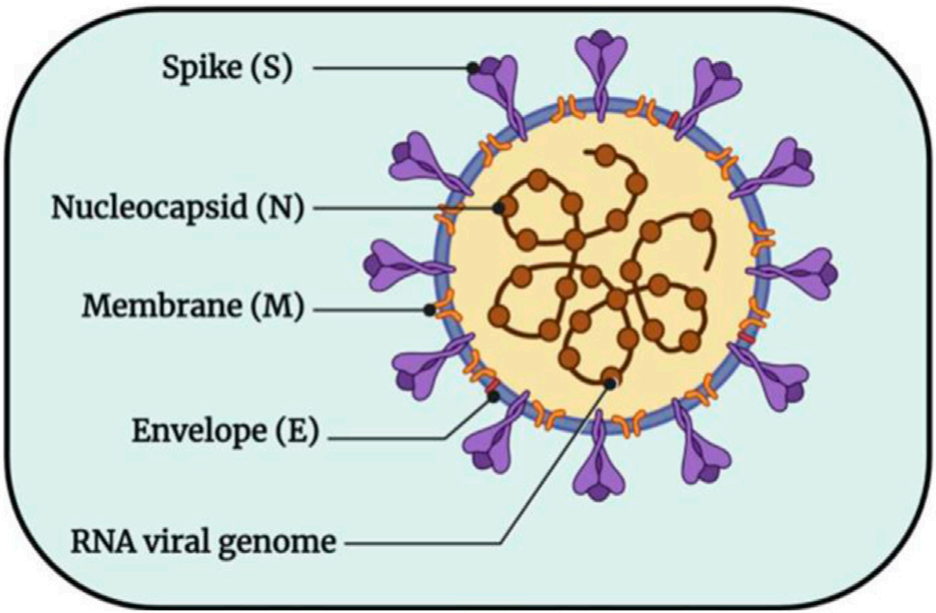
The structural pattern diagram of SARS-CoV-2 (Chitranshi et al., 2020).

The key to responding to this outbreak is to analyze SARS-CoV-2 at the molecular level, to fundamentally elucidate the pathogenic mechanism of the virus and the binding process of the virus and host cells, and to take timely and effective preventive and therapeutic countermeasures (Wang and Zhang, 2020). SARS-CoV-2 particles are visibly round or oval, and their diameter is between 60–140 nm, because their surface is uneven and their appearance is shaped like a crown, so it is called coronavirus. As shown in Figure 1 (Chitranshi et al., 2020), its surface is surrounded by lipid membranes, Spike protein (S), Membrane protein (M), Envelope protein (E), and Nucleocapsid protein (N). Some studies have shown that with the exact infection mechanism as SARS-CoV, SARS-CoV-2 also uses the spike glycoprotein on its surface to bind to receptors on the surface of host cells and then enter cells through endocytosis (Imai et al., 2005; Zhou et al., 2020). Therefore, the S protein somewhat determines the host’s range. M proteins, E proteins and N proteins are attached to host receptors, bind to host nucleocapsid proteins, and play roles in multiple processes such as the assembly and release of viral genes, which is the key for viruses to attach to host receptors and enter target cells, that is, the key to the pathogenic mechanism of viruses After the virus enters the cell, it immediately releases its protein coat and single-stranded RNA encoding its genetic material. The released RNA is immediately bound to the ribosome in the host cell and translated into functional proteins necessary for its replication, and these proteins include main protease (M^pro^), 3-chymotrypsin-like protease (3CL^pro^), Pain-like protease (PL^pro^) and RNA-dependent RNA polymerase (RdRp) (Ibrahim et al., 2021). Among them, M^pro^ generates proteases necessary for subsequent viral RNA replication by promoting the cleavage of polyproteins to ensure the transcription and replication of viral RNAs in host cells and finally release them outside the host cells (Zhang et al., 2020; Das et al., 2021; Khan et al., 2021). Through this pathogenic process, treating patients can be achieved by inhibiting the production of proteases required for the virus’s replication.

In general, all proteases that play a role in the viral life cycle can serve as targeting proteins for antiviral drugs, but by contrast, the M^pro^ plays an indispensable role (Mehmood et al., 2022). Among the many coronaviruses studies, M^pro^ is currently the most studied protease target (Adem et al., 2022). M^pro^ is fully conserved in all released SARS coronavirus genome sequences, highly homologous to M^pro^ of other coronaviruses, and has no human homolog (Amin et al., 2021; Pelly and Liotta, 2021; Qiao et al., 2021). Many other M^pro^ inhibitors of coronaviruses can be used to study M^pro^ inhibitory activity against SARS-CoV-2, some of which are being tested clinically. If some show activity against SARS-CoV-2, they could be rapidly developed against SARS-CoV-2 drugs. The three-dimensional crystal structure of M^pro^ has been analyzed. Based on its highly conserved three-dimensional structure, compounds with potential inhibitory effects on M^pro^ can be obtained by virtual screening of pharmaceutical databases (Li et al., 2022). Therefore, SARS-CoV-2 M^pro^ is a crucial target for structure-based anti-SARS-CoV-2 drug design.

Computer-aided drug design (CADD) has been widely used in predicting drug-target interactions and evaluating drug safety (Sabe et al., 2021). By simulating the interaction between the compound and the target protein by computer, the compound molecules that have specific effects on the target protein can be screened from thousands or even tens of thousands of drug molecules. On the one hand, the fortuity in traditional experiments is excluded, and on the other hand, the efficiency of the experiment is greatly improved. It is possible to obtain results that cannot be obtained in traditional experimental analysis, thereby improving clinical efficiency and production efficiency for drug discovery and development, significantly saving time, labor, and money. During epidemics, drug repurposing by testing broad-spectrum drugs already used for other coronavirus infections is a fast and feasible approach (Hung et al., 2020). The research in this paper mainly uses CADD, combined with the compounds that have been proven to be inhibitory to M^pro^, based on the three-dimensional structure of M^pro^, to screen and design potential compound molecules with inhibitory activity to SARS-CoV-2 M^pro^. This article targets SARS-CoV-2 M^pro^ and uses Discovery Studio 2020 (DS 2020) to achieve high-throughput molecular docking. Pharmacophore models of compounds with inhibitory effects on major proteases were constructed, and candidate compounds with inhibitory activity against SARS-CoV-2 M^pro^ were identified through virtual screening of databases and molecular docking.

In conclusion, because of the current critical situation of the COVID-19 epidemic, this study provides scientific theoretical guidance for researching specific anti-SARS-CoV-2 drugs by studying specific targeted inhibitors of the SARS-CoV-2, combined with the CADD method. This paper closely combines the development direction of the discipline, focuses on solving the problems that people are urgently concerned about, and provides a theoretical basis for the research and development of new coronavirus drugs, which are of great significance to the healthy development of human beings.

## 2 Materials and methods

### 2.1 Protein target preparation

First, the researchers prepared the X-ray crystal structure of the M^pro^ (PDB ID: 7BE7) (Costanzi et al., 2021). We downloaded the protein three-dimensional crystal structure with good resolution (1.68 Ǻ) from the protein database (RCSB) (http://www.pdbus.org). Target protein M^pro^ was pretreated with “Clean Protein” and “Prepare Protein” in DS 2020. The protein structure’s water molecules and small ligand molecules were removed, and a three-dimensional model of the protein without redundant ligands was obtained.

### 2.2 Quantitative structure-activity relationship (QSAR) analyses

#### 2.2.1 Data collection and arrangement

After reviewing the literature and sorting the database, 48 known M^pro^ inhibitors were collected in this study. According to these compounds’ structure and inhibitory activity values, these compounds are divided into two sets: the training set and the test set. The training set contains 30 compounds, and the test set contains 18 compounds, the training set is shown in Supplementary Figures S2–S4, and the test set is shown in Supplementary Figures S5, S6. In order to produce an excellent quantitative pharmacophore model, the training set and test set compounds must adhere to the following rules (Golbraikh et al., 2003): 1) Compounds should be distributed across different orders of magnitude. Moreover, the compounds of each order magnitude were for at least 3; 2) Compounds in the same order of magnitude should be structurally diverse; 3) The activities of molecules in similar structures should differ by at least an order of magnitude; 4) Compounds contained “Activ” and “Uncert” values, with the structures and active values in the training and testing sets being very similar to one another (Sakkiah et al., 2012).

In this study, the active range of the training set compounds was between 0.0138 μM and 53.00 μM (0.0138 μM < IC_50_ < 53.00 μM), spanning four orders of magnitude; the active range of the test set was between 0.20 μM and 28.10 μM (0.20 μM < IC_50_ < 28.10 μM), spanning three orders of magnitude, and the number of compounds on each order of magnitude of both sets was more than three, strictly adhering to the above rules.

#### 2.2.2 Data preprocessing

The experimenter uses the software BIOVIA Draw 2020 to map the 2D structure of the compounds in the training and test sets as preparation files for calculations. The prepared files are then imported into the software Discovery Studio 2020 (DS 2020) to convert the 2D structure of the compound into a 3D structure. Insert the attributes “Activ” and “Uncert” in the table browser. “Activ” is the active value of the compound, which can be an IC_50_ value or a Ki value, and in this experiment the IC_50_ value of the compound is used. The “Uncert” value is set uniformly to 1.5 for all compounds. After the above preparations, perform the following operations on the compound: 1) Prepare ligand molecules using the “Prepare or Filter Ligands” module in the DS software: Small Molecules→Prepare or Filter ligands→Prepare ligands. The parameters are set as follows, Change Ionization: False; Generate Tautomers: False; Generate Isomers: False; Fix Bad Valencies: True; 2) Small molecule structure optimization: Small Molecules→Minimize Ligands→Full minimization. In this study, the PDB database was used to find the best inhibitor MG-132 (28) currently bound to M^pro^, and used this as a control to find key amino acid residues.

#### 2.2.3 Pharmacophore model establishment

The “3D Quantitative Structure-Activity Relationship (QSAR)” module in the software DS2020 can build a pharmacophore model with activity prediction based on the structure of the reported compounds with clear activity values. The algorithm first constructs an initial pharmacophore model that can share active molecules and cannot share inactive molecules and then further optimizes the model by simulated annealing. The resulting model can predict the activity of compounds and guide the optimization of compounds to improve their activity. The training set was selected to construct the pharmacophore model, and the compounds in the training set had a clear activity value (IC_50_) for M^pro^, and the following operations were performed.

According to the pharmacophore construction algorithm, the top two compounds with the highest activity ranking are defined as the active compounds, and the algorithm is “MA × Unc^MA^ - A/Unc^A^ > 0.0,” and the two rows represented by them are displayed in light green. The lowest compound is defined as an inactive compound, and the algorithm is “log(A)—log(MA) > 3.5,” and the rows it represents are shown in light pink. The “A” represents the active value of the compound, and the “MA” represents the activity value of the most active compound.

The characteristic elements of the pharmacophore are then determined according to the “Feature mapping” module, targeting the light green compound, which is the top two compounds in this experiment. This calculation process can identify the possible location of the characteristic element in the two compounds. The results showed that both compounds contained five characteristic elements: Hydrophobe, Donor, Acceptor, Ionizable Positive and Ring Aromatic. Hydrophobic Aromatic was used instead of Ring Aromatic when building pharmacophore models because the former has fewer restrictions, only defines one site, and does not have the plane and vector limitations of the latter. In the 3D QSAR module, the pharmacophore model is constructed, the parameters of Maximum Conformations are set to 255 and the parameters of Energy Threshold are set to 10. The above parameters represent a conformational space in which up to 255 conformations are generated for each small molecule to characterize small molecules, where only conformations with energy values within the energy threshold of 10 kcal/mol are retained. Select Hydrophobe, Donor, Acceptor, Ionizable Positive, and Hydrophobic Aromatic in the Select Features column.

#### 2.2.4 Pharmacophore model selection and validation

After constructing the pharmacophore, the researchers obtained a total of 10 models. DS2020 will give these 10 models a ranking. Usually, the first place in the overall ranking of pharmacophores is also the best. However, rankings are only determined by cost value, so the top-ranked pharmacophore model is not the best in some exceptional cases. This requires a comprehensive analysis of parameters such as “Features,” “Total cost” and “Correlation”. The most important thing is to use the test set molecules with known activity values to verify whether the predictive ability of this pharmacophore model for the activity values of molecules other than the training set meets the expected requirements. Therefore we performed a quadruple validation method in this experiment to find the best pharmacophore model. The first validation is Root mean square deviation (RMS), Correlation coefficient and cost difference (△cost); The second validation is Fischer’s randomization test; The third validation is the activity verification of the test set; The fourth validation is the Heat map of Ligand profiler.

### 2.3 Establishment of database and virtual database screening

Virtual database screening can be used to effectively discover potential small molecule compounds with higher activity, which may have significant inhibitory activity on the target protein, and these compounds are all validated drug small molecules (Rohilla et al., 2017; Zhang et al., 2017; Kaushik et al., 2018). After full validation, researchers will obtain the best pharmacophore model. The researchers performed a virtual database screening based on the pharmacodynamic profile, active site, and various parameters of the best pharmacophore model. In this study, we selected the Traditional Chinese Medicine, Druglike, and MiniMaybridge databases, which included 51,564, 5,384, and 2,000 compounds, respectively.

In order to make the resulting compound more likely to become a drug, the researchers also test the properties of the compound according to the Lipinski’s “rule of 5” principle, removing those molecules that are not suitable for becoming drugs, thereby narrowing the scope of the screening (Valko and Reynolds, 2005; Tang et al., 2012). Compounds that comply with the Lipinski’s “rule of 5” principle have better pharmacokinetic properties, and they will exert higher bioavailability in the organism’s metabolism process. Hence, they are also more likely to be oral drugs and worthy of structural modification and other more in-depth studies.

### 2.4 Molecular docking

Molecular docking analysis can predict the affinity of small molecule compounds to target proteins by using a series of biological, mathematical and computer-based models, allowing researchers to evaluate the interaction between compound molecules (drug molecules) and proteases through specific binding sites mutual lease (Gupta et al., 2018). Zev et al. (2021) evaluated several leading docking programs in a study: Glide, DOCK, AutoDock, AutoDock Vina, FRED, and EnzyDock. In this study, Dev rated the molecular docking ability of these programs. The criteria are whether the docking procedure can correctly identify the binding pattern of ligands and M^pro^, and whether it can accurately and objectively score the docking results. In the overall success of all projects, the top three are as follows, Glide and EnzyDock reproduce the correct crystal structure pose (rmsd <2 Å) for over 50% of the structures, with success rates of 64% and 70%, respectively, while for AutoDock, this rate falls to 40%. After a comprehensive analysis, we completed the molecular docking of compounds with M^pro^ through CDOCKER and verified the docking results through AutoDock (G et al., 1990; Morris et al., 1996; Morris et al., 2009).

We first adopted the CDOCKER molecular docking strategy. CDOCKER is a molecular docking method based on the CHARMm force field, which can produce high-precision docking results. The selected compounds were pretreated with “prepare or Filter Ligands” and “Minimization of Ligands,” and the processed compounds were directly docked with the target proteins. We used the crystal structure of M^pro^ obtained in the RCSB Protein database with a resolution of 2.16 Ǻ, pre-processed the target protease through the “Protein Prepare,” defined the receptor binding site and prepared the docking system. Firstly, the From Receptor chamber function in the Definie Site toolbar was used to search for the cavity in the receptor as a possible binding site. Based on Grid Search and the “eraser” algorithm, we define possible binding sites in the receptor by looking for cavities. A total of nine possible binding sites were found. This was followed by searching the PDB database for small molecules bound to M^pro^ conformations. The two were analyzed comprehensively and finally identified possible binding sites. Molecular docking of small molecules with proteins is carried out at this site. After selecting the appropriate binding site, the pretreated small molecule ligand and receptor introduction procedure are performed for molecular docking.

The researchers analyzed the molecular docking results of the CDOCKER and screened out the 10 most suitable compounds. The researchers used AutoDock to reconnect 10 compounds to ensure the study’s rigor.

AutoDock is a free and widely used molecular docking software. AutoDock uses “rapid grid-based energy evaluation” and “efficient search of torsional freedom” methods to make the calculation results as accurate as possible while reasonably balancing the use of computing resources. Import the selected optimal compounds and M^pro^ into AutoDock software to complete the preparation of the world file. AutoGrid budgets the affinity for each atomic type in the ligand molecule. AutoDock then completes molecular docking between the compound and the acceptor. Finally, AutoDockTools was used to analyze the molecular docking results.

### 2.5 Molecular dynamic simulation

Molecular dynamics (MD) simulation is a rapid development of molecular simulation methods in recent years. It is based on classical mechanics, quantum mechanics, and statistical mechanics. It uses computer numerical methods to solve the equations of motion of molecular systems to simulate and study the structure and properties of molecular systems (Wu et al., 2022). This technique can obtain the motion trajectory of atoms and observe various microscopic details in the process of atomic motion (Childers and Daggett, 2023). It is a powerful complement to theoretical calculations and experiments. This study subjected the best-selected compound to MD simulations to simulate the interaction between the ligand and M^pro^. MD simulations were performed with AMBER18 using the ff14SB force field. The force field parameters of inhibitors were built by the Antechamber module of AMBER18 (Wang et al., 2006; Lu et al., 2023). In the initial system, remove non-inhibitor molecules and water molecules outside the 5 Å range and add missing hydrogen atoms through the Leap module (Yamashita, 2023). Three chloride ions were added using the leap module in AMBER based on a coulomb potential grid to keep the system electrically neutral. TIP3P explicit water boxes with an 8.0 Å distance around the solute were added to these complexes. The system’s energy is minimized, and the process is divided into two parts: the steepest descent method and the conjugate gradient method. The solvent and ions were subjected to 12,000 steps of steepest decent minimization followed by 8,000 steps of conjugate gradient minimization with the protein and small molecules fixed with a 500 kcal mol^−1^ Å^−2^ constraint. Then, each system was totally minimized for another 20,000 steps with no restraint (12,000 steps of steepest decent minimization and 8,000 steps of conjugate gradient minimization). After minimization, the three systems were heated up gradually from 0 to 310 K in the NVT ensemble, applying harmonic restraints with a force constant of 10.0 kcal mol^−1^ Å^−2^ on the protein and small molecules. A Langevin thermostat was adopted. NPT constant voltage operating balance with 500 ps at 310 K constant voltage balance. Then these systems went through 500 ps equilibrium MD simulations. Finally, a total of 200 ns was simulated for each system under NPT ensemble conditions with the cut-off at 10 Å. The time step was set to 2 fs. The researchers then conducted Root-mean-square deviation (RMSD) and Root-mean-square fluctuation (RMSF) studies and performed energy calculations.

## 3 Results

### 3.1 Analysis and validation of pharmacophore models

The 3DQSAR module in DS software generates 10 pharmacophore models by analyzing and calculating the characteristics of 30 training set compounds (Table 1). There are three characteristic elements in these 10 pharmacophore models, namely, hydrogen bond acceptor (HBA), hydrophobic aromatic (HR) and hydrophobic (HY). The Cost attribute is considered the most efficient way to select the best pharmacophore model, with△Cost (Null cost—Total cost) representing the probability of true correlation of the data. At the same time, parameters such as Features, RMS and Correlation also have a certain degree of reference value. After comprehensively analyzing various parameters, the researchers assumed that the fifth pharmacophore model was the best model. Because the fifth pharmacophore has the most features element sites (HBA, HBA, HR, HY), the RMS (3.07) and Correlation (0.79) are excellent, and the gap between the △Cost (235.19) and the first-ranked pharmacophore is also within the acceptable range.

**TABLE 1:** 10 pharmacophore models generated by M^pro^ inhibitors through the. HypoGen module.

Hypo	Total cost	Cost difference^a^	Error	RMS^b^	Correlation	Features
No.						
1	132.30	337.14	115.59	1.65	0.95	HBA,HR,HY
2	132.36	337.07	115.65	1.65	0.95	HBA,HR,HY
3	147.60	321.84	129.80	1.91	0.93	HBA,HR,HY
4	197.71	271.73	181.86	2.65	0.85	HBA,HR,HY
5	234.25	235.19	219.71	3.07	0.79	HBA,HBA,HR,HY
6	237.32	232.12	222.75	3.10	0.79	HBA,HR,HY,HY
7	238.31	231.13	222.96	3.11	0.79	HBA,HBA,HR,HY
8	240.22	229.22	225.72	3.14	0.78	HBA,HBA,HR,HY
9	240.48	228.96	225.99	3.14	0.78	HBA,HBA,HR,HY
10	240.56	228.88	222.07	3.14	0.78	HBA,HBA,HR,HY

^a^ Cost difference between the null and the total cost, null cost = 469.44, fixed cost = 87.85, for the Hypo6 weight = 1.87, configuration cost = 13.35.

^b^ RMS, root mean square deviation.

^c^ HBA, hydrogen bond acceptor; HY, hydrophobic.

**FIGURE 2:**
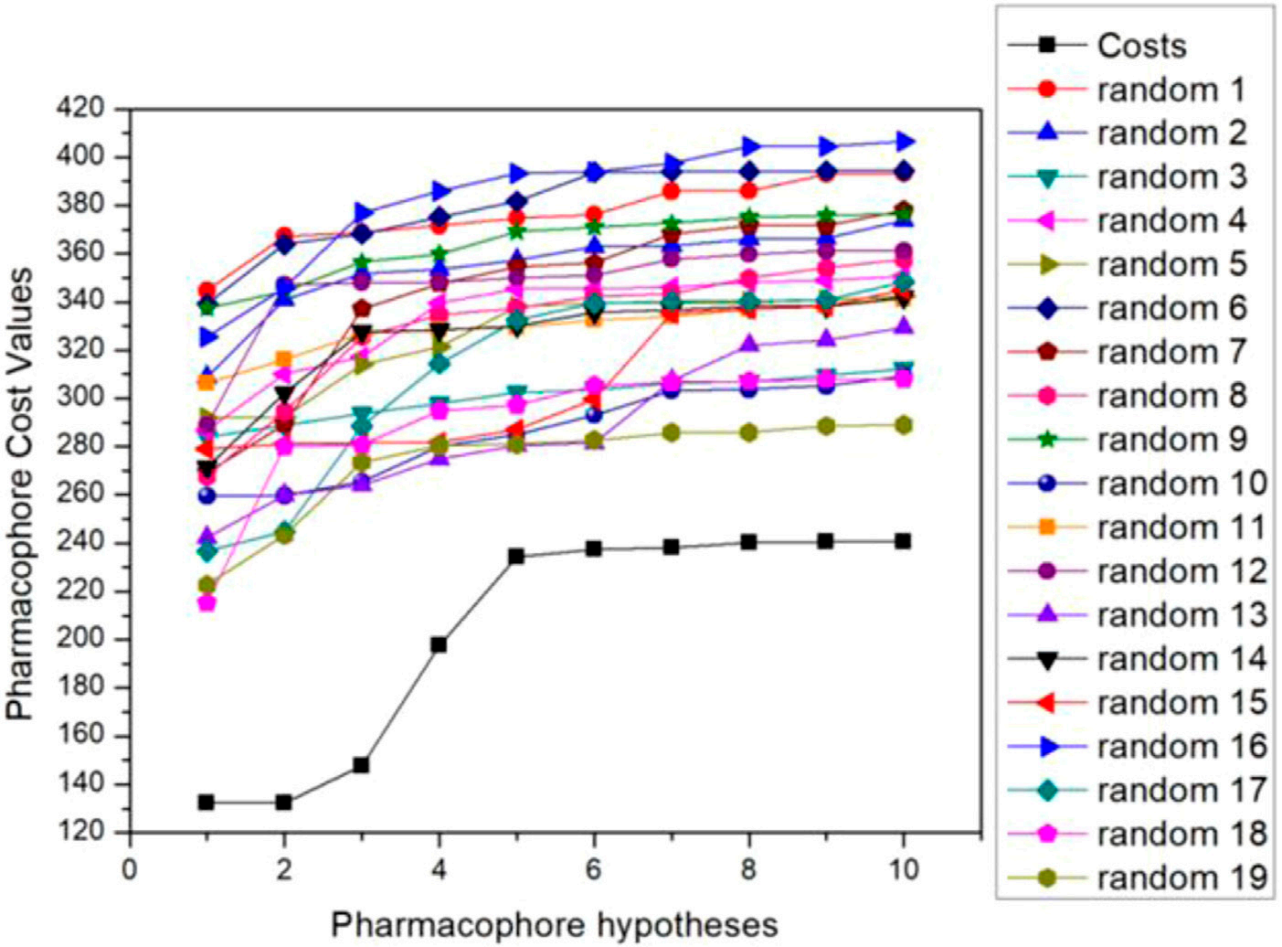
Fischer validation: the total cost of the initial hypothesis (Hypo5) and the 19 random spreadsheets on the 95% confidence level.

The researchers then validated the optimal pharmacophore model selected. Fischer’s randomization test is a way to verify statistical confidence in pharmacophore models through a Catscramble module inside Catalyst. This method aims to randomly scramble the activity values of all training sets and achieve a 95% confidence level to generate 19 random pharmacophore models. The results of Fischer verification are shown in Figure 2. As the figure shows, our hypothetical pharmacophore Hypo5 performs best compared to the generated 19 pharmacophore models, and the total cost value of the original hypothesis is significantly lower than the randomly generated 19 pharmacophores. This also shows that the cost difference of the original hypothesis is higher than that of the randomly generated 19 pharmacophore models, thus proving that our hypothesis that the proposed model 5 is the best pharmacophore model is correct.

The training set contains 30 M^pro^ inhibitors and the test set contains 18 M^pro^ inhibitors, which are analyzed to test the predictive power of the best pharmacophore model Hypo5. Tables 2, 3 show the experimental and predicted activity values of the training and test set compounds based on the pharmacophore model 5, respectively. Supplementary Figures S6, S7 show the correlation between the experimental (logIC_50_) and predictive activity (logEstimate) of the pharmacophore model 5 for training and test set compounds.

**TABLE 2:** Experimental and estimate activity [IC_50_ (µM)] evaluation of the training sets based on the pharmacophore model Hypo5.

Compound no.	Fit value^a^	Exp.IC_50_ µM	Estimate	Error	Experimental scale^b^	Estimated scale^b^
1	6.12	0.01	0.14	2.16	+++	+++
2	4.43	0.06	0.95	3.12	+++	+++
3	5.38	0.07	0.78	−1.26	+++	+++
4	5.32	0.20	0.88	−1.07	+++	+++
5	6.64	0.31	0.04	3.14	+++	+++
6	6.04	0.95	0.17	−1.18	+++	+++
7	6.52	0.98	0.06	−1.14	+++	+++
8	4.47	4.82	6.35	−6.30	++	+++
9	4.48	5.00	6.20	−6.29	++	+++
10	4.48	6.80	6.19	−5.98	++	+++
11	4.41	7.20	6.34	−3.81	++	+++
12	4.48	8.30	6.19	−4.04	++	+++
13	4.41	9.10	7.34	−2.04	++	+++
14	4.46	9.40	6.44	−2.33	++	+++
15	4.48	10.00	6.21	−2.09	++	+++
16	4.48	10.00	6.25	−1.70	++	+++
17	4.46	10.00	6.52	−1.99	++	+++
18	4.41	10.00	7.25	−1.38	++	+++
19	4.53	13.00	5.54	−1.80	++	+++
20	4.47	13.00	6.26	−1.60	++	+++
21	4.48	15.00	6.25	−1.46	++	+++
22	4.41	15.00	7.19	−1.31	++	+++
23	4.87	21.70	6.25	4.23	++	+++
24	4.48	25.00	6.21	−1.34	++	+++
25	4.43	28.00	6.87	−1.05	++	+++
26	4.45	37.00	6.60	−1.03	++	+++
27	5.14	39.00	7.35	−3.70	++	+++
28	4.65	50.00	7.17	1.10	++	+++
29	4.23	23.80	11.05	−4.53	++	++
30	4.37	30.60	8.06	−1.24	++	++

^a^ Fit value represents the degree of overlap between the features in Hypo1 and the chemical features in the molecule.

^b^ Activity scale: IC_50_ < 1 μM = +++ (highly active); 1 μM ≤ IC_50_ < 100 μM = ++ (moderately active); IC_50_ ≥ 100 μM = + (low active).

**TABLE 3:** Experimental and estimate activity [IC_50_ (µM)] evaluation of the test sets based on the pharmacophore model Hypo5.

Compound no.	Fit value^a^	Exp.IC_50_ µM	Estimate	Error	Experimental scale^b^	Estimated scale^b^
−1	6.04	0.20	0.17	1.18	+++	+++
−2	6.40	0.23	0.07	1.13	+++	+++
−3	5.40	0.98	0.75	−1.31	+++	+++
−4	4.58	6.20	4.92	−1.26	++	++
−5	4.58	6.90	4.97	−1.39	++	++
−6	4.48	9.19	6.20	−1.48	++	++
−7	4.78	10.00	5.07	−0.26	++	++
−8	4.48	10.00	6.23	−1.61	++	++
−9	4.47	10.00	6.30	−1.59	++	++
−10	4.48	11.00	6.19	−1.78	++	++
−11	4.44	12.00	6.78	−1.77	++	++
−12	4.46	13.00	6.52	−1.99	++	++
−13	4.47	14.00	6.26	−2.23	++	++
−14	4.48	15.00	6.24	−2.40	++	++
−15	4.48	20.00	6.19	−3.23	++	++
−16	4.47	24.14	6.32	−3.82	++	++
−17	4.48	28.00	6.19	−4.52	++	++
−18	4.48	28.10	6.21	−4.52	++	++

^a^ Fit value represents the degree of overlap between the features in Hypo1 and the chemical features in the molecule.

^b^ Activity scale: +++ highly active (<1 μM), ++ moderately active (1–100 μM) and + weakly active (>100 μM).

In Tables 2, 3, we are divided into three levels according to the activity values (IC_50_) of the training and test sets: IC_50_ < 1 µM = +++ (high activity); 1 µM ≤ IC_50_ < 100 µM = ++ (moderate activity); IC_50_ ≥ 100 µM = + (low effective or ineffective activity). In the prediction of the activity values of compounds in Table 2, it can be seen that the experimental and predicted activities of compounds 1–7 are both shown as high activity, the experimental activity of compounds 8–30 is moderate, while the predicted activity of compounds 8–28 is still showing high activity, and only the predicted activity of compound 29–30 is moderate. In Table 3, both the experimental and predicted activities of compounds 1–3 showed high activity, and compounds 4–18 showed moderate activity, which was completely consistent with the predicted results. Although the predictive activity of the training set compounds 8–28 is higher than the experimental activity, it is still in line with our predicted results, which shows that our prediction results are correct. In addition, the experimental and predictive activity regression analysis of the training and test set compounds gave excellent correlation coefficients (*R* ^2^) of 0.841 and 0.754, respectively, as shown in Supplementary Figures S8, S9.

The validation of the best pharmacophore 5 is based on the “Ligand Profiler” module, which superimposes compound molecules in the training set with the key chemical characteristics of the pharmacophore. By executing the “Ligand Profiler,” the results will be displayed as a heat map. As shown in Supplementary Figures S6, S7, the abscissa represents the 10 validated pharmacophore models, and the ordinate represents the compounds in the training or test set. The overlapping matching of the pharmacodynamic characteristics and key chemical bonds of the compound and the pharmacophore is represented by the color of different cells, and when the color of the lattice is closer to red, the better the match between the compound and the pharmacophore. As can be seen from the figure, the seventh pharmacophore in the training and test sets matches the compound well, and the fifth pharmacophore is not the best performer. However, all things considered, it is still considered that the fifth pharmacophore still meets our original hypothesis.

By performing quadruple validation of the best pharmacophore model 5, we find that the pharmacophore model 5 is consistent with the predicted results in these quadruple validations. This thoroughly verifies the rationality of the best pharmacophore of the original hypothesis as model 5, thus laying a foundation for the scientific nature of subsequent theoretical research.

### 3.2 Virtual screening results

The verified optimal pharmacophore model was successfully used for virtual screening of the Traditional Chinese Medicine, Druglike Diverse, and MiniMaybridge databases. These compounds are then subjected to Lipiniski’s “rule of five” and less active compounds are removed, leaving compounds with IC_50_ values below 1 μM. Up to now, 257 compounds with good activity have been obtained for follow-up work.

### 3.3 Molecular docking analysis

**FIGURE 3:**
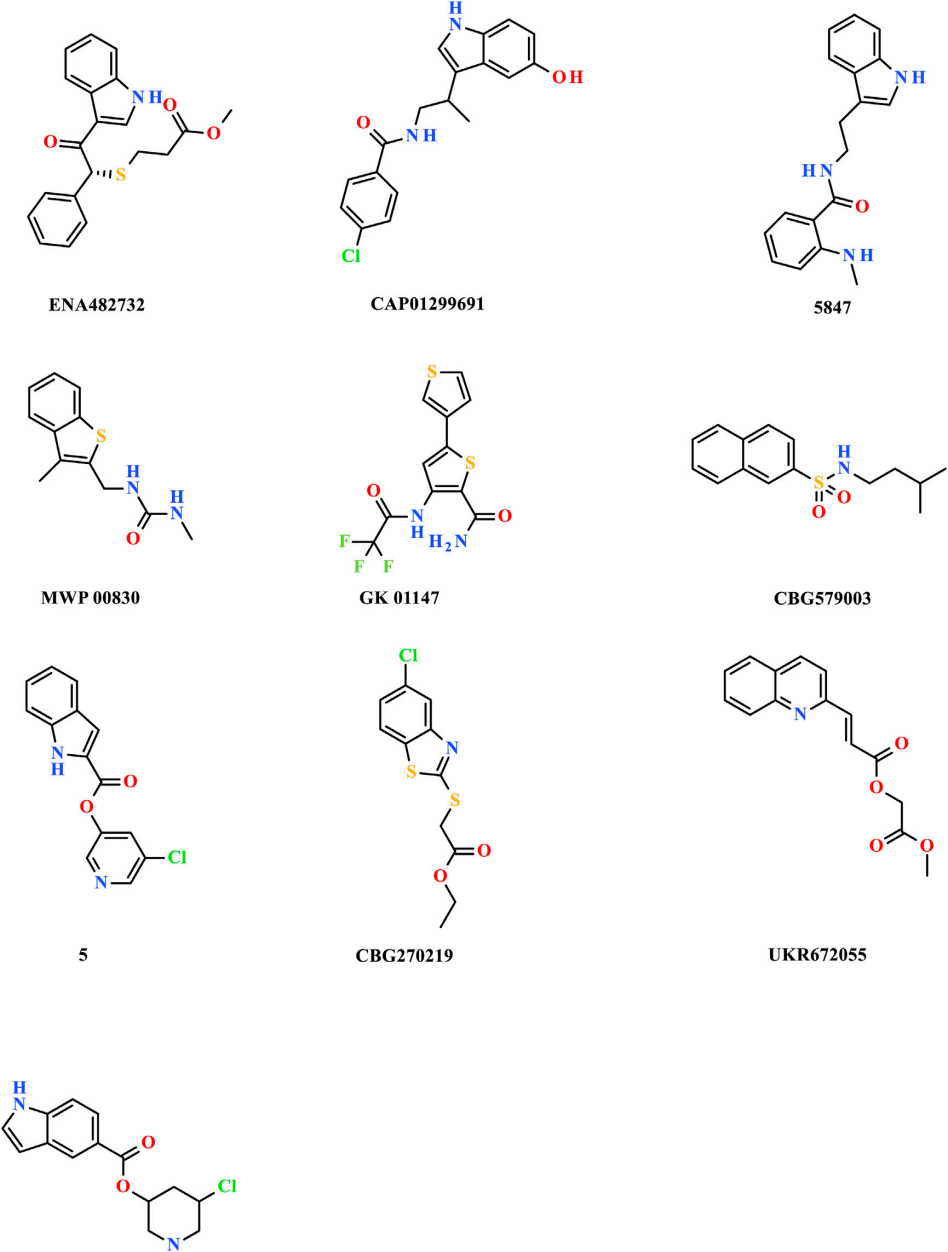
The structure of the top 10 compounds.

**FIGURE 4:**
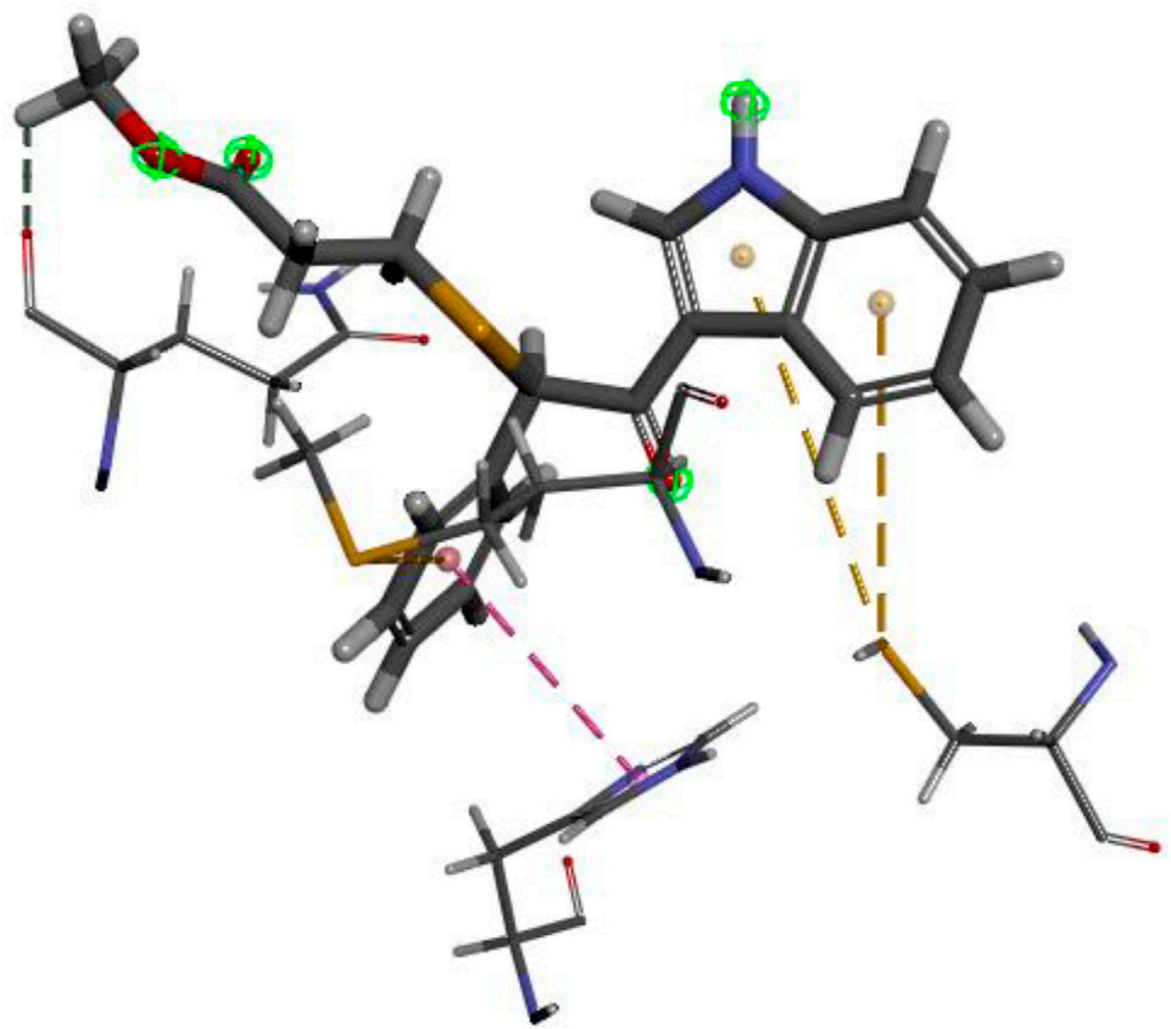
The docking interactions of compound ENA482732 with M^pro^.

**FIGURE 5:**
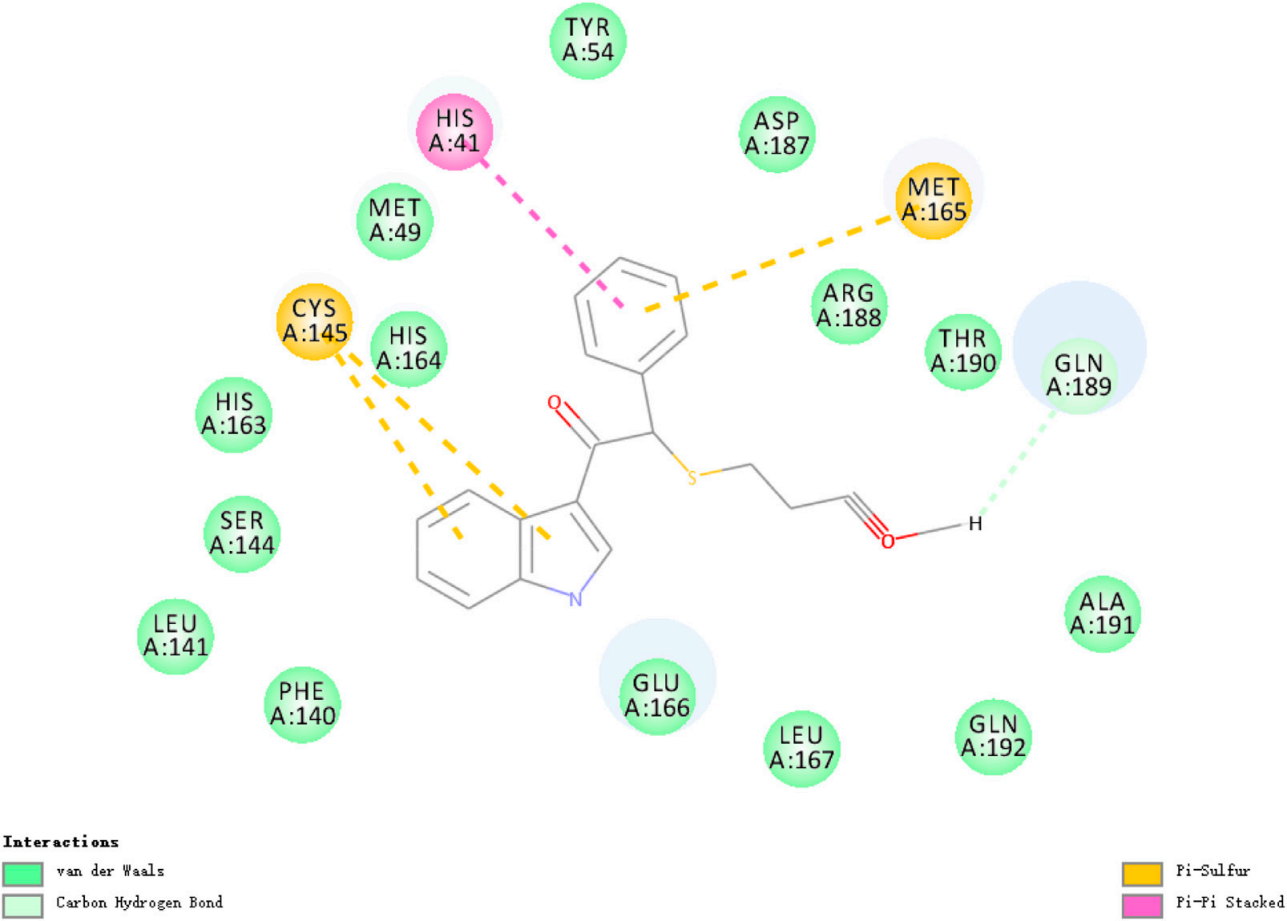
2D analysis of non-bonded interaction between compound ENA482732 and M^pro^.

The researchers carried out molecular docking with M^pro^ on the final 257 compounds screened. The researchers selected the top 10 compounds for comparative analysis with MG-132, and the docking results are shown in Tables 4, 5. The structure of the top 10 compounds is shown in Figure 3. We conducted ADMET studies on the best ten compounds; the results are presented in Table 6. Table 6 shows that the top 10 compounds have good intestinal absorption, water solubility, and blood-brain barrier penetration. In Table 4, the CDOCKER_INTERACTION_ENERGY (CIE) represents an estimate of ligand-receptor interaction energy, and CDOCKER_ENERGY (CE) considers the ligand’s strain the ligand when placed within the active site of the same compound. We can see that whether it is CE ranking or CIE ranking, compound ENA482732 ranks first. So we assume that the compound ENA482732 is the best candidate compound. Table 5 shows the interacting amino acids in which the top ten ligands dock with the molecule of interest, highlighting the amino acids consistent with MG-132 in bold. After analyzing the interacting amino acids of the top ten compounds and the control MG-132, HIS41, CYS145, and GLN189 were important residues for M^pro^ inhibition activity. At the same time, in Table 5, the researchers can also clearly observe that the compound ENA482732 interacts with M^pro^ in much more amounts of amino acids than other compounds. The researchers then focused on the molecular docking results of the compound ENA482732 and M^pro^. The non-bonding interaction between the acceptor and ligand is represented using different color scales during docking. Figures 4, 5 show four types of interactions between compound ENA482732 and the target protein M^pro^, including Van der Waals, Pi-Sulfur, Carbon Hydrogen Bond, and Pi-Pi Stacked. The Pi-Sulfur interaction occurs between the phenyl of compound ENA482732 and methionine 165 (MET: 165) of M^pro^. The indole group of the compound also has a Pi-Sulfur interaction with cysteine at 145 (CYS: 145). There was a Pi-Pi Stacked interaction between the phenyl group of the compound and histidine at site 41 (HIS: 41) of the receptor. The Carbon Hydrogen Bond occurs between the hydrogen atoms of the compound and glutamate at site 189 (GLN: 189). The compound also forms hydrophobic interactions with the remaining adjacent amino acids. This further proves our hypothesis that the compound ENA482732 is the best candidate and has great potential to be developed as a highly effective inhibitor.

**TABLE 4:** Results from molecular docking of M^pro^ inhibitors.

Compound	CDOCKER_ENERGY	CDOCKER_INTERACTION_ENERGY
ENA482732	32.98	41.69
CAP01299691	29.88	35.96
5487.cdx	27.07	36.75
MWP 00830	25.90	30.77
GK 01147	25.86	31.58
CBG579003	24.10	35.90
5	22.41	32.96
CBG270219	21.28	30.57
UKR672055	20.68	30.01
8	20.52	31.40

**TABLE 5:** The interaction amino acid in the ligand-protein for the top 10 docking compounds including the control compound.

Compound	Interaction acids
ENA482732	HIS41, MET49, TYR54,PHE140, LEU141, SER144, CYS145, HIS163, HIS164, MET165, GLU166, LEU167, ARG188, GLN189, THR190, ALA191, GLN192
CAP01299691	LEU27, HIS41, CYS145, MET165, GLU166, GLN189
5487.cdx	MET49, ASN142, GLY143, HIS163, MET165, HIS172, GLN189
MWP 00830	HIS41, MET49, TYR54, HIS164, MET165, ASP187
GK 01147	HIS41, CYS145, MET165, GLU166
CBG579003	HIS41, MET49, MET165, GLU166, GLN189
5	MET49, GLN189
CBG270219	LEU27, HIS41, MET49, CYS145
UKR672055	MET49, ASP187
8	LEU27, HIS41, MET49, TYR54, CYS145, MET165, ASP187
MG-132	ASN28, HIS41, GLY143, CYS145, SER147, HIS164, GLU166, PRO168, GLN189

**TABLE 6:** The ADMET property of 7 compounds.

Compound	ADMET-absorption-level^a^	ADMET-solubility	ADMET-solubility-level^b^	ADMET-BBB	ADMET-BBB-level^c^
ENA482732	0	−3.926	3	0.031	1
CAP01299691	0	−4.752	2	−0.051	2
5487.cdx	0	−3.808	3	−0.13	2
MWP 00830	0	−3.308	3	−0.126	2
GK 01147	0	−4.329	2	−0.703	3
CBG579003	0	−4.317	2	0.094	1
5	0	−4.782	2	0.053	1
CBG270219	0	−4.808	2	0.504	1
UKR672055	0	−3.411	3	−0.397	2
8	0	−3.283	3	−0.426	2

^a^ When ADMET_Absorption_T2_2D < 6.1261 (inside 95%), the Level is 0, which has good absorption. ADMET_Absorption_T2_2D is the Mahalanobis distance for the compound in the ADMET_PSA_2D, ADMET_AlogP98 plane. It is referenced from the center of the region of chemical space defined by well absorbed-compounds.

^b^ When −6.0 <ADMET-Solubility < −4.1, the Level is 2, which has lower water solubility. When −4.1 <ADMET-Solubility < −2.0, the Level is 3, which has good water solubility.

^c^ When Level 1, the Brain-Blood ratio between 1:1 and 5:1 indicates the high probability of the drug passing through the blood-brain barrier. The larger the level, the lower the probability of crossing the blood-brain barrier.

**FIGURE 6:**
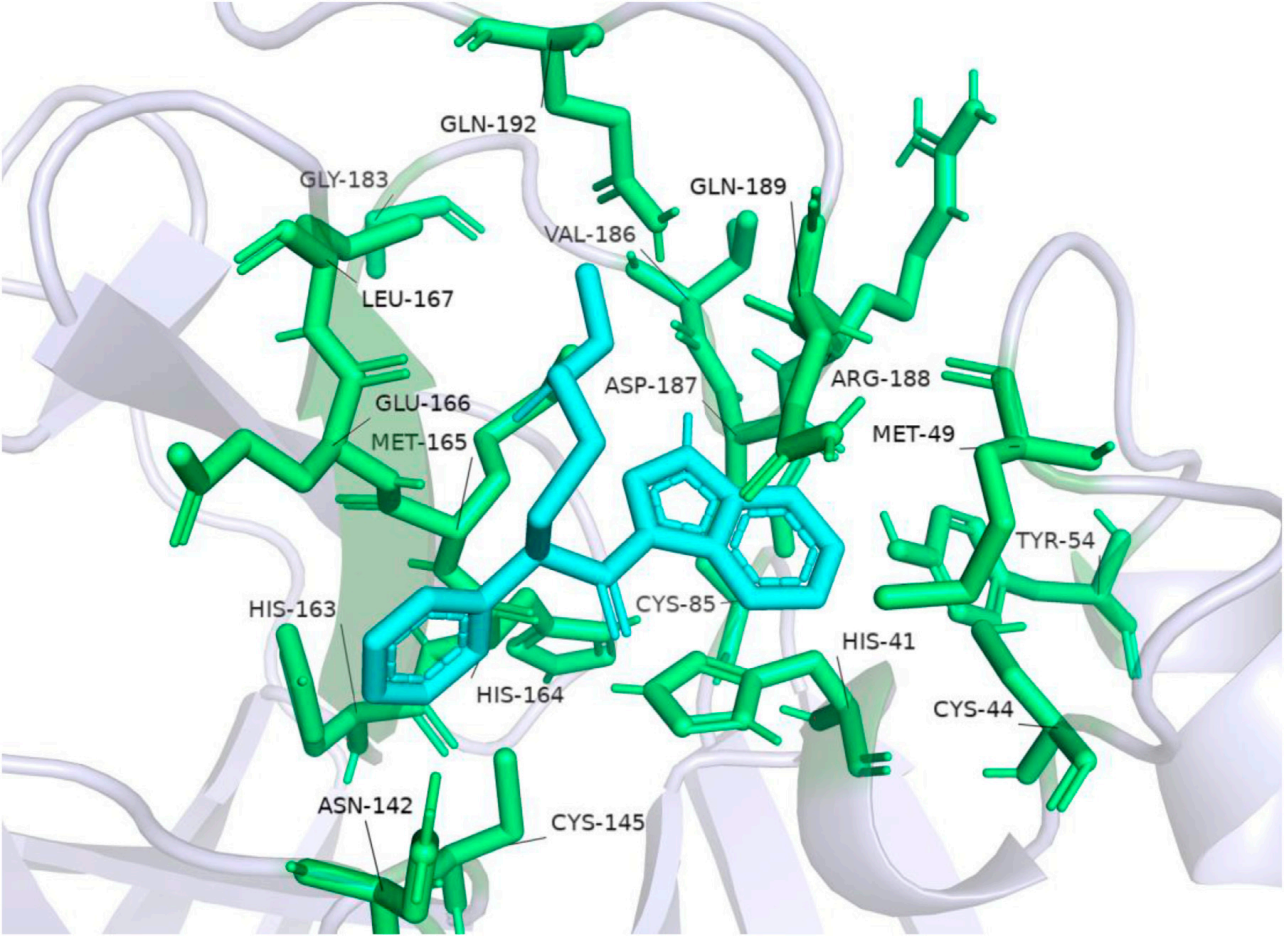
The result of molecular docking of compound ENA482732 and M^pro^ by AutoDock.

The accuracy of the above results was verified by the molecular docking of the compound ENA482732 to M^pro^ by AutoDock, and the results are shown in Figure 6. The binding energy of the compound ENA to the receptor is −6.15 kcal, and the efficiency of the ligand is −0.25. The phenyl group of compound ENA482732 interacts with cysteine at site 145 (CYS: 145) of the receptor protein. The indole group of the compound has a Pi-Cation interaction with histidine at site 41 (HIS: 41) and Pi-Sulfur interaction with cysteine at site 44 (CYS: 44). In addition, the indole group also has an Alkyl interaction with methionine at the site 49 (MET: 49) and site 165 (MET: 165), and it forms a conventional hydrogen bond with arginine at site 188 (ARG: 188). From this result, the indole group of the compound ENA482732 plays a vital role in improving the inhibitory activity, providing a theoretical basis for modifying subsequent compounds. The AutoDock results are similar to CDOCKER, and both demonstrate that ENA482732 can stably bind to M^pro^ receptors.

In this study, CDOCKER was first used for molecular docking to screen out the best compound ENA482732. The researchers then used AutoDock to verify the binding stability of the compound ENA482732 to M^pro^. Both docking methods clearly show that the ENA482731 screened by the compound can be stably bound to M^pro^.

### 3.4 Analysis of molecular dynamics results

**FIGURE 7:**
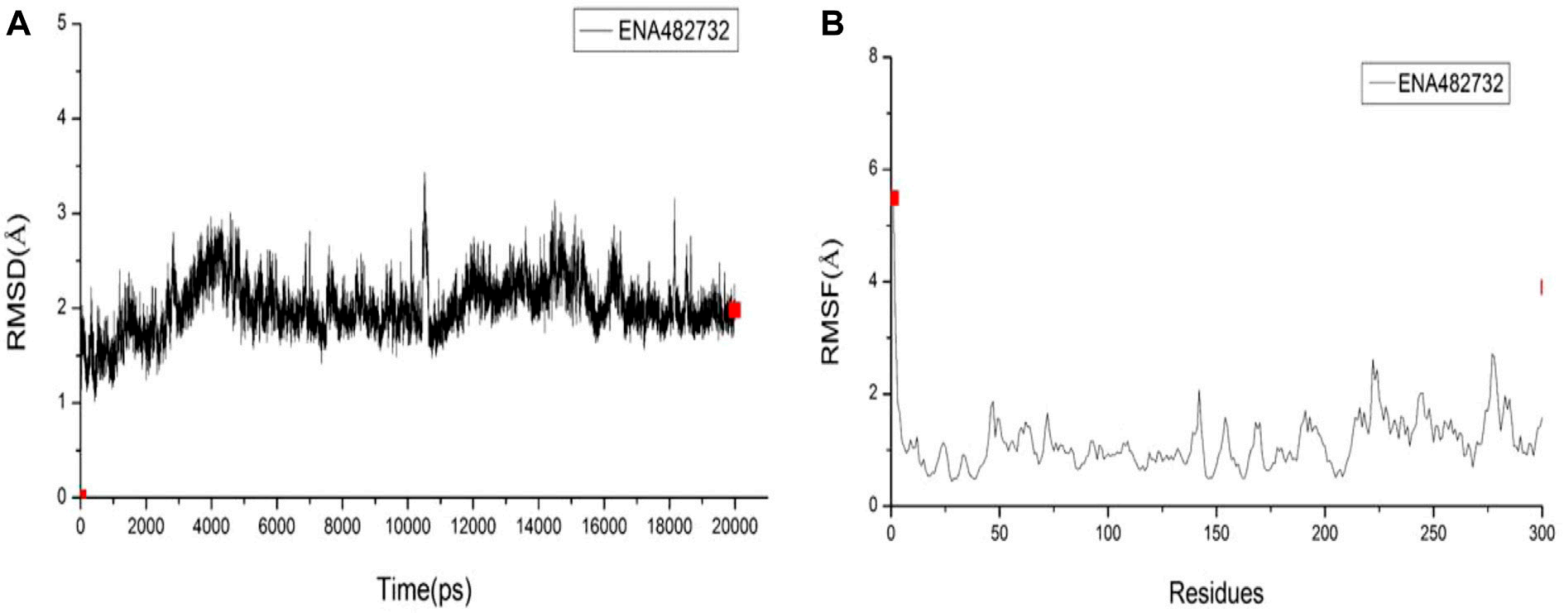
The RMSD and RMSF results of compound ENA482732. **(A)** RMSD. **(B)** RMSF.

The best-selected compound, ENA482732, was subjected to molecular dynamics simulations. RMSD is a detection method that can provide a sketch of the conformational changes by comparing changes in the positions of the atoms with a reference structure. In Figure 7A, it can be seen that the fluctuation range of the compound ENA482732 is within 2 Å, the fluctuation amplitude is weak, and the linear relationship tends to converge, which indicates that the complex remains stable throughout the simulation time. RMSF is a curve that can offer details on fluctuations of each residue over the simulation time. A high RMSF value represents that the certain residue has a large flexibility, while a low one manifests large stability. The RMSF values are displayed in Figure 7B. The residues with a high value were checked, only to find that most of these residues are located on the edge of the complex and far away from the inhibitor binding pocket. The residues by ligand to M^pro^ are HIS41, MET49, TYR54, PHE140, LEU141, SER144, CYS145, HIS163, HIS164, MET165, GLU166, LEU167, ARG188, GLN189, THR190, ALA191, GLN192, which have very low RMSF values and strong stability. Combined with the calculation of binding free energy, Binding Energy is −15.2 kcal/mol, and Entropic Energy is 20.0 kcal/mol. The above data indicate that the compound ENA482732 can bind stably to M^pro^. Compound ENA482732 has great potential to be developed as a potent inhibitor.

## 4 Discussion

Since the outbreak of the COVID-19, anti-SARS-CoV-2 drugs targeting M^pro^ have been continuously reported and many have entered clinical studies. However, only Paxlovid developed by Pfizer and Xocova of Shionogi Pharmaceutical Company has been approved for marketing. Therefore, safe, reliable and broad-spectrum novel M^pro^ inhibitors are still an urgent clinical need at present and in the future.

During this outbreak, researchers have realized the analysis of the virus sequence quickly and further functional and structural studies, which have provided a powerful scientific and technological force for controlling COVID-19. The study of virus-related biology, especially the crystal analysis of the structure, has promoted the research of M^pro^ as a therapeutic strategy and the development of targeted inhibitors. In this study, we explored the structural characteristics of M^pro^, whose gene sequence set is completely conserved and has no human homologous genes, which is well suited as a target for drug treatment. Pharmacophore design and virtual screening were used to find compounds that have inhibitory activity against M^pro^ and have the potential to be developed as drugs. In order to ensure that we screen out compounds with inhibitory activity against M^pro^ in the small molecule database, firstly, based on the efficacy fragments or pharmacodynamic characteristics of the active compounds we have, the existing pharmacophore integration methods are used to retain the effective pharmacodynamic characteristics, remove the pharmacodynamic characteristics and ineffective characteristics that cause adverse reactions to design the pharmacophore model for M^pro^. In order to ensure the rationality of the pharmacophore model, we conducted four verifications, and the results showed that the designed pharmacophore model could accurately screen out compounds with inhibitory activity against M^pro^. The activity of the screened compounds was tested by molecular docking of the CDOCKER program, and the results were verified with AutoDock. This ensures this study’s rigor and scientific nature and that the screened compounds have inhibitory solid activity against M^pro^ and good pharmacokinetic properties.

After analyzing the selected compounds, it was found that the first compound, ENA482732, was very much in line with our expectations. Many binding sites and various intermolecular interaction forces ensure the stable binding of compound ENA482732 to acceptors. At the same time, we found that in the structure of M^pro^, the cavity formed by HIS41, CYS145, and GLN189 as the main sites is very suitable for binding small molecule inhibitors. The structure of the compound ENA482732 is simple and clear, and it is convenient for subsequent structural optimization to improve its inhibitory activity further. Studies of its structure also tell us that the indole group can interact with M^pro^ in various ways. This suggests that known small molecule inhibitors can be modified to add indole groups in appropriate locations to improve their stability in binding to M^pro^.

This study only provides theoretical research for the development of M^pro^ inhibitors. Molecular dynamics confirmed that the screened compounds had inhibitory solid activity against M^pro^, but it was still theoretical. The development of formal inhibitors is still some way off. In future studies, the theory will be extended to experiments through enzyme activity experiments to verify further whether the compound has inhibitory activity. Preclinical safety, pharmacodynamics, and pharmacy studies are conducted after ENA482732 or modified compounds are identified as drug candidates. This is used to observe the biological activity of the compound against the target disease and evaluate the compound’s safety to support the initiation of clinical trials.

## 5 Conclusion

Although the large-scale outbreak has ended, there are still variants of SARS-CoV-2 that cause small-scale infections. Mutated strains are characterized by increased transmission, infectivity, and reduced virulence. M^pro^ is an indispensable functional protein for viral replication and is highly conserved compared to other proteases. Therefore, the compounds screened in this study have therapeutic effects on subsequent infections of SARS-CoV-2 variants. This article will increase people’s understanding of COVID-19, provide a new perspective for the research of anti-SARS-CoV-2 drugs, and promote the development of new SARS-CoV-2 inhibitors.

## Data Availability

The datasets presented in this study can be found in online repositories. The names of the repository/repositories and accession number(s) can be found in the article/Supplementary Material.
