# Supplementary material for: Computer-aided drug design for virtual-screening and active-predicting of main protease (Mpro) inhibitors against SARS-CoV-2
Source: Front Pharmacol. 2023 Nov 7;14:1288363. doi: 10.3389/fphar.2023.1288363 (PMC10661973; doi:10.3389/fphar.2023.1288363)
Supplement: Supplementary file 1 [file Table1.DOCX]

Supplementary Material

Article Title

Renhui Dai, Hongwei Gao*, Ruiling Su

*** Correspondence:** Corresponding Author: gaohongw369@ldu.edu.cn

## Supplementary Figures


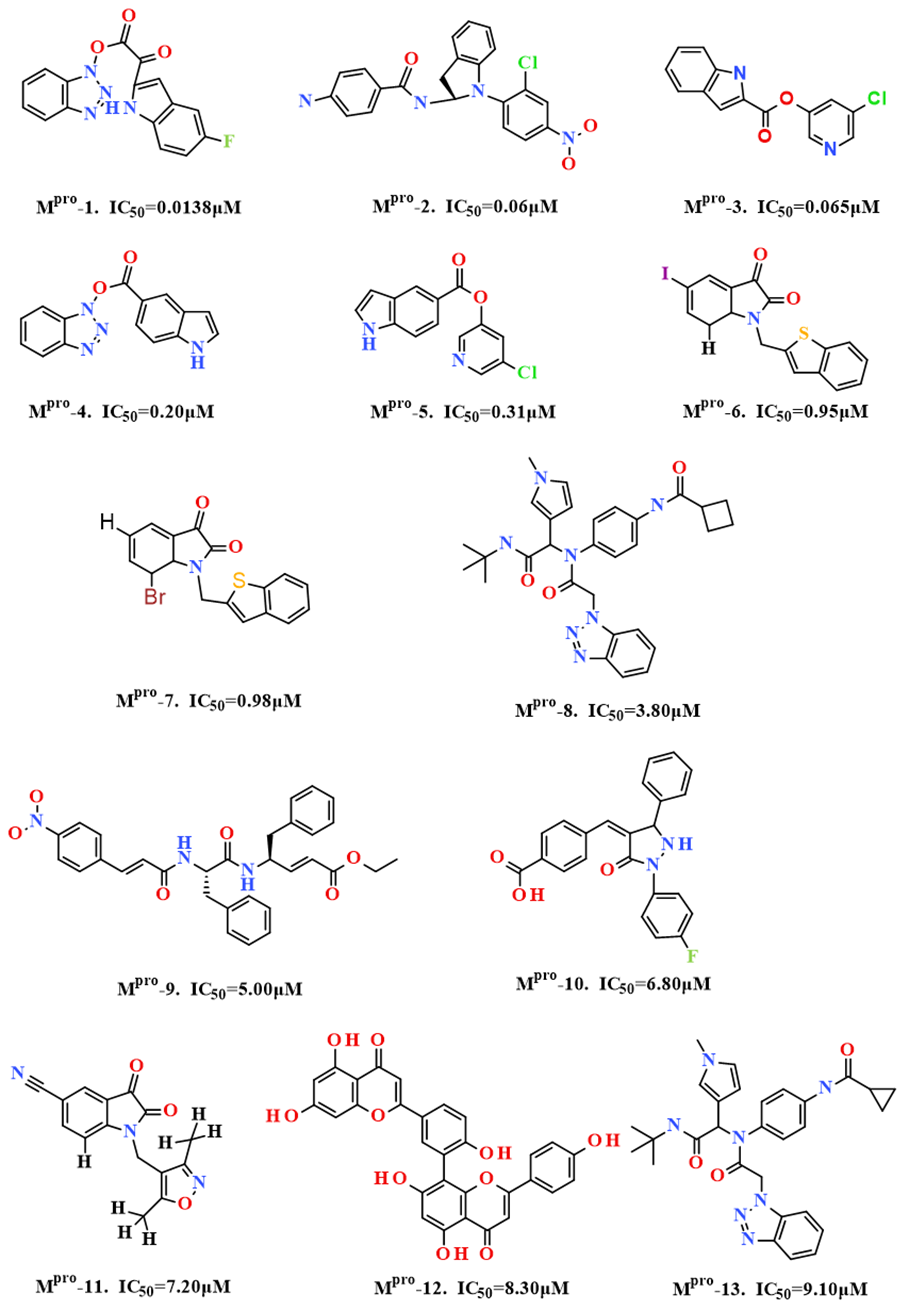


**Supplementary Figure 1.** Structure and biological activity of Compounds 1-13 in the training set (IC50, μM).

**
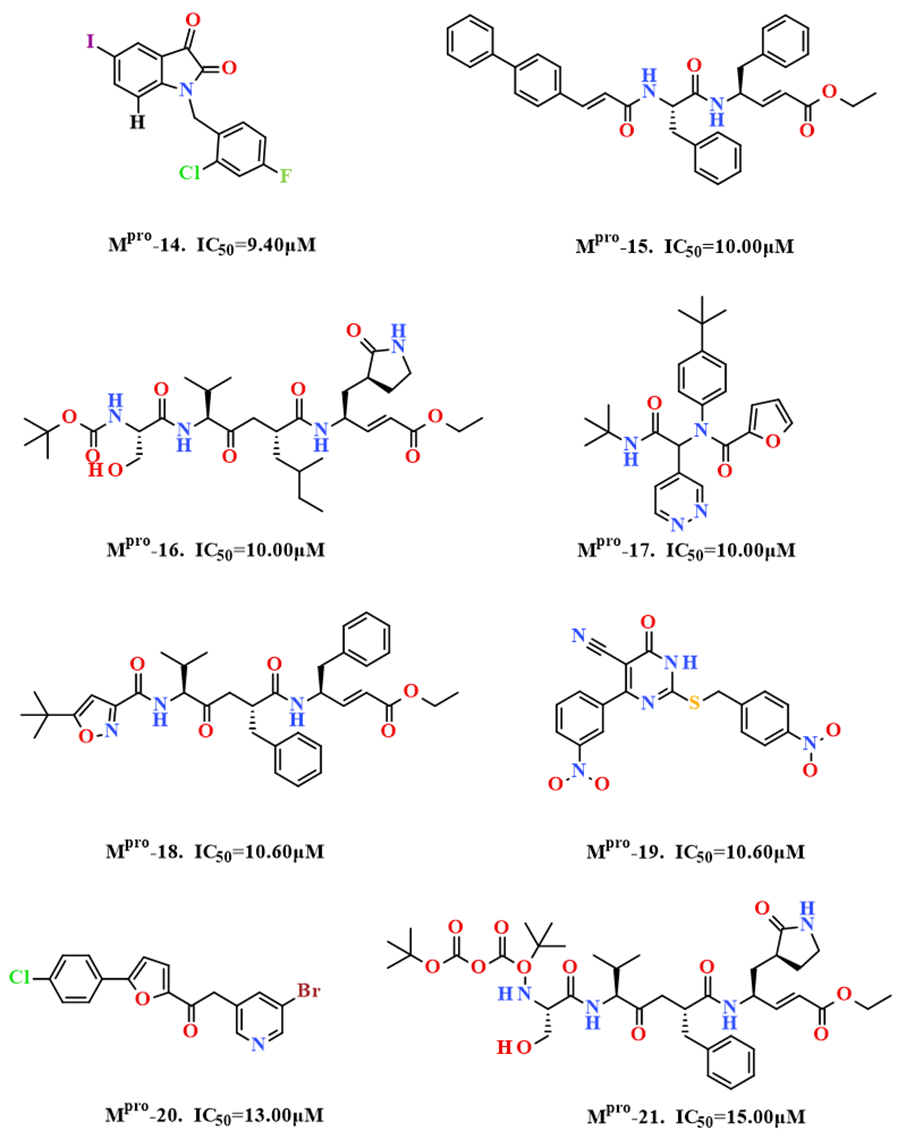
**

**Supplementary Figure 2.** Structure and biological activity of Compounds 14-21 in the training set (IC_50_, μM).

**
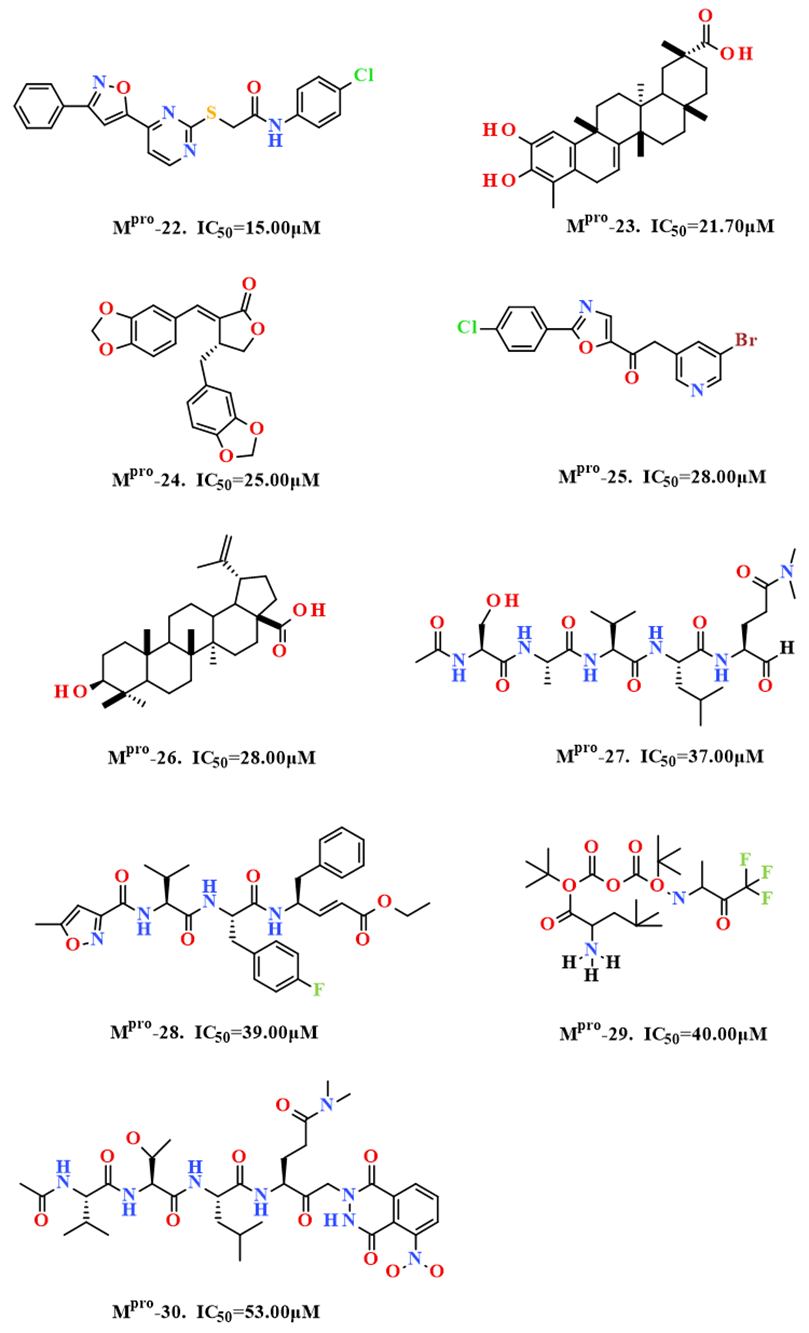
**

**Supplementary Figure 3.** Structure and biological activity of Compounds 22-30 in the training set (IC_50_, μM).

**
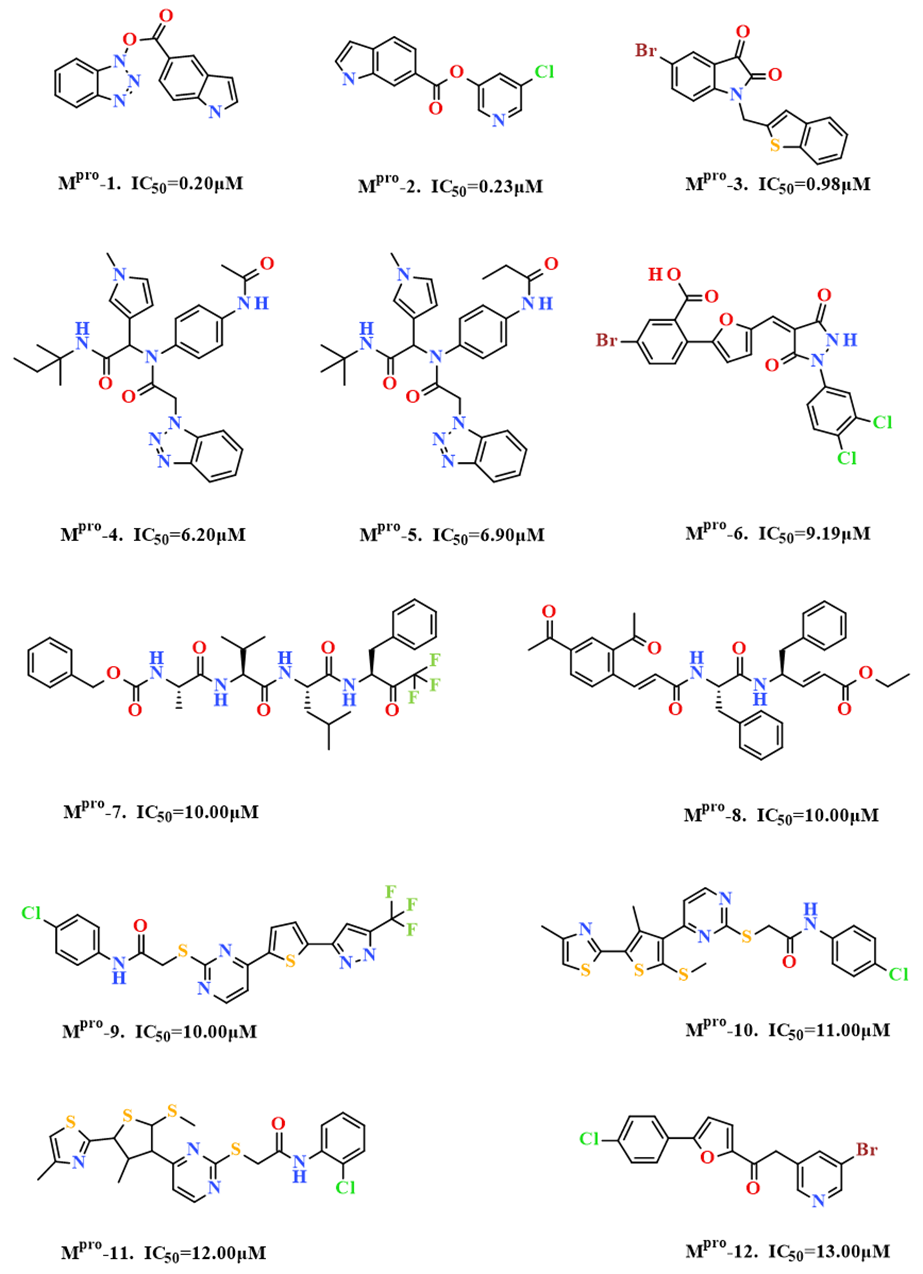
**

**Supplementary Figure 4.** Structure and biological activity of Compounds 1-12 in the test set (IC_50_, μM).

**
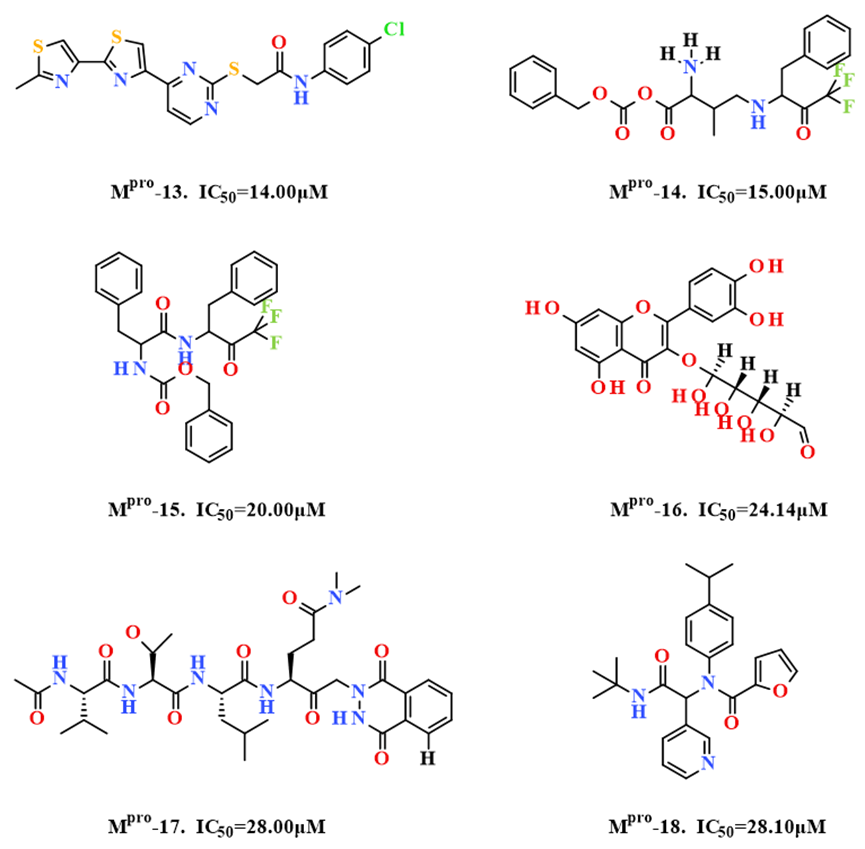
**

**Supplementary Figure 5.** Structure and biological activity of Compounds 13-18 in the test set (IC_50_, μM).


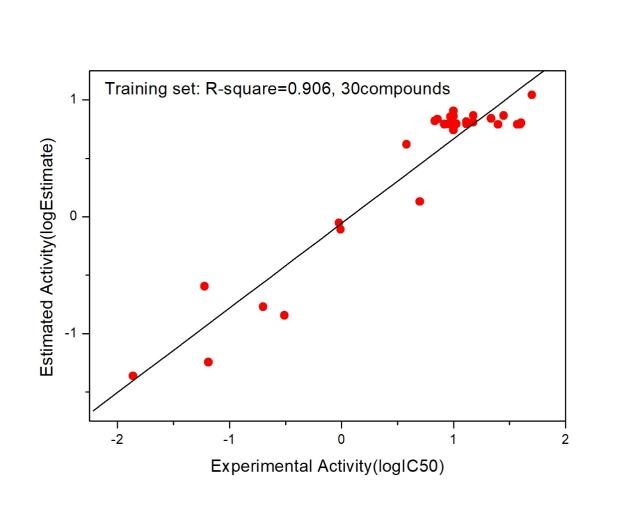


**Supplementary Figure 6.** Correlation between the experimental and estimated activity (logIC_50_) by Hypo5 for the training set compounds.


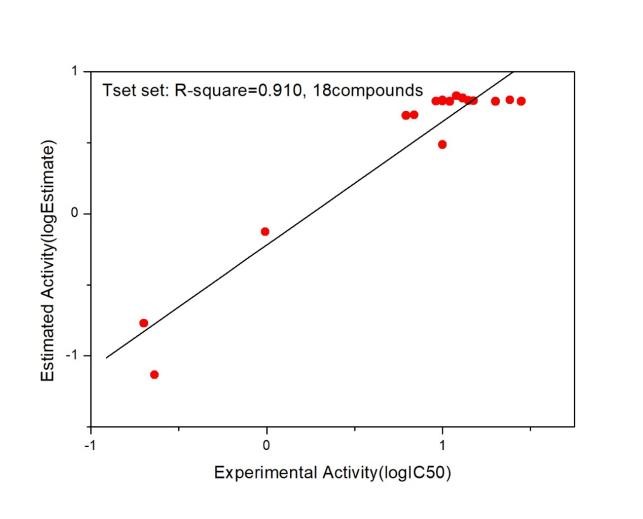


**Supplementary Figure 7.** Correlation between the experimental and estimated activity (logIC_50_) by Hypo5 for the test set compounds.

**
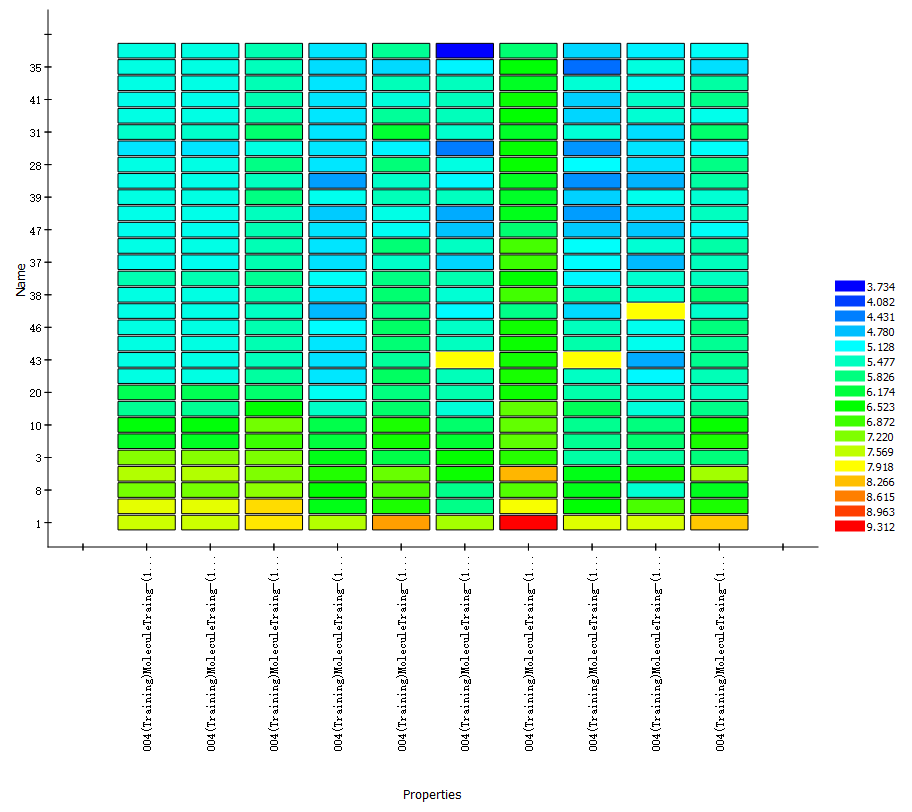
**

**Supplementary Figure 8.** Heat map of "Ligand Profiler" predicted by 10 Hypos for the training set compounds.

**
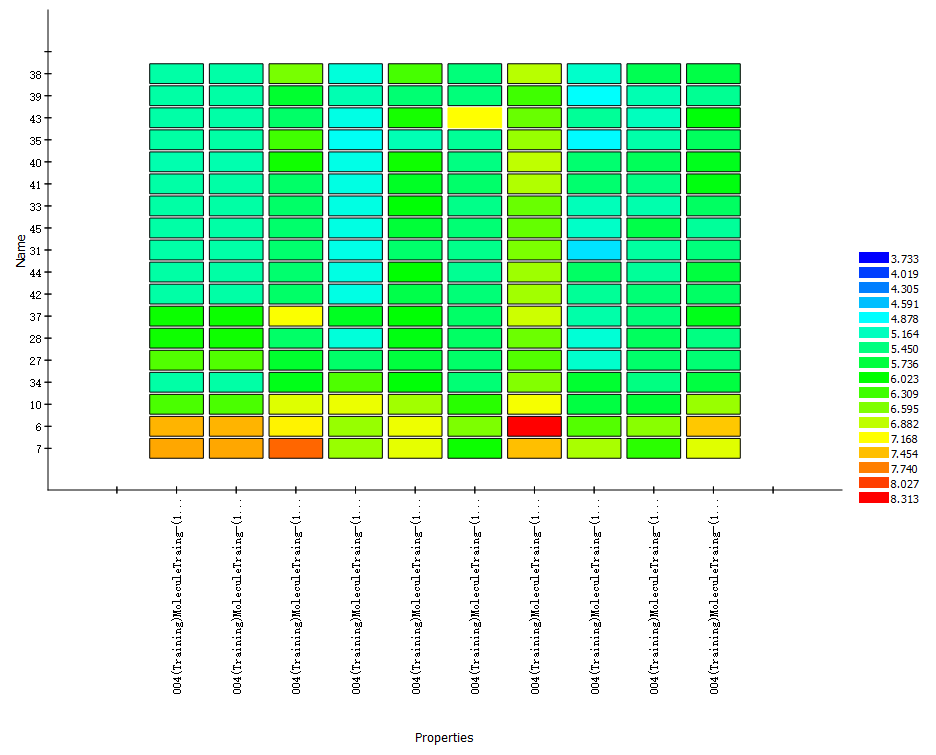
**

**Supplementary Figure 9.** Heat map of "Ligand Profiler" predicted by 10 Hypos for the test set compounds.
